# SARS-CoV-2 infection prevention and control measures in Belgian schools between December 2020 and June 2021 and their association with seroprevalence: a cross-sectional analysis of a prospective cohort study

**DOI:** 10.1186/s12889-023-15806-5

**Published:** 2023-05-16

**Authors:** Milena Callies, Ines Kabouche, Isabelle Desombere, Joanna Merckx, Mathieu Roelants, Melissa Vermeulen, Els Duysburgh

**Affiliations:** 1 Department of Epidemiology and Public Health, Sciensano, Brussels, Belgium; 2 Department of Infectious Diseases in Humans, Immune Response, Sciensano, Brussels, Belgium; 3 Department of Epidemiology, Biostatistics and Occupational Health, McGill University, Montreal, QC Canada; 4 Environment and Health, Department of Public Health and Primary Care, University of Leuven, Louvain, KU Belgium

**Keywords:** Infection prevention and control, School-aged children, School staff, Seroprevalence, SARS-CoV-2, Belgium

## Abstract

**Background:**

To protect school-aged children from the potential consequences of a new viral infection, public health authorities recommended to implement infection prevention and control (IPC) measures in school settings. Few studies evaluated the implementation of these measures and their effect on SARS-CoV-2 infection rates among pupils and staff. The aim of this study was to describe the implementation of infection prevention and control (IPC) measures in Belgian schools and assess its relation to the prevalence of anti-SARS-CoV-2 antibodies among pupils and staff.

**Methods:**

We conducted a prospective cohort study in a representative sample of primary and secondary schools in Belgium between December 2020 and June 2021. The implementation of IPC measures in schools was assessed using a questionnaire. Schools were classified according to their compliance with the implementation of IPC measures as ‘poor’, ‘moderate’ or ‘thorough’. Saliva samples were collected from pupils and staff to determine the SARS-CoV-2 seroprevalence. To assess the association between the strength of implementation of IPC measures and SARS-CoV-2 seroprevalence among pupils and staff, we conducted a cross-sectional analysis using the data collected in December 2020/January 2021.

**Results:**

A variety of IPC measures (ventilation, hygiene and physical distancing) was implemented by more than 60% of schools, with most attention placed on hygiene measures. In January 2021, poor implementation of IPC measures was associated with an increase in anti-SARS-CoV-2 antibody prevalence among pupils from 8.6% (95%CI: 4.5 – 16.6) to 16.7% (95%CI: 10.2 – 27.4) and staff from 11.5% (95%CI: 8.1 – 16.4) to 17.6% (95%CI: 11.5 – 27.0). This association was only statistically significant for the assessment of all IPC measures together in the population comprised of pupils and staff.

**Conclusions:**

Belgian schools were relatively compliant with recommended IPC measures at the school level.

Higher SARS-CoV-2 seroprevalence among pupils and staff was found in schools with poor implementation of IPC measures, compared to schools with thorough implementation.

**Trial registration:**

This trial is registered under the NCT04613817 ClinicalTrials.gov Identifier on November 3, 2020.

**Supplementary Information:**

The online version contains supplementary material available at 10.1186/s12889-023-15806-5.

## Background

To protect the global population, including school-aged children, against the spread of SARS-CoV-2, national and regional authorities implemented infection prevention and control (IPC) measures in various societal settings. Because the role of school-aged children in the transmission dynamics was unclear, IPC measures against COVID-19 were implemented in schools from an early stage [1]. The assumption that school-aged children play a major role in maintaining the epidemic, as is the case for seasonal influenza, made many governments implement drastic measures such as school closures with a switch to remote learning [2–4]. The United Nations Educational, Scientific and Cultural Organization (UNESCO) reported that 190 countries closed their schools by April 2020 which affected 90% of the world’s school-aged population [5, 6]. At that time, the implementation of these measures was criticized due to the lack of evidence on the impact of SARS-CoV-2 transmission among children and youth, with many highlighting that these measures could cause additional mental and socio-economic problems among school-aged children [2]. In response, many European countries reopened their schools and implemented a variety of IPC measures at school level with the objective to create the safest possible environment for in-person teaching. However, the implementation of IPC measures differed nationally, regionally and across educational levels [1].

International organizations (WHO, UNESCO, UNICEF, IFRC) and national public health authorities recommended a broad range of COVID-19 IPC measures for primary and secondary educational settings [7]. The classification of these measures changed over time and differed across organizations and national entities [7–11]. For example; WHO defined the following categories of measures: 1. personal protection, 2. environmental, 3. physical distancing, and 4. screening [7].

In Belgium, several IPC measures were recommended in schools which can be classified into the above-mentioned WHO categories. From mid-March until May 2020, remote learning was mandatory for all schools. Schools reopened for in-person teaching in May 2020 while implementing IPC measures such as hand and respiratory hygiene, ventilation and physical distancing measures and introducing an intensive contact tracing programme. Furthermore, the last four grades of secondary school (ages 14–17) were required to organize 50% of classes online in order to limit physical presence at school. Mask wearing was compulsory for all primary and secondary school staff and all pupils from secondary schools [12].

Studies examining the impact of IPC measures in schools remain limited [13]. Due to the complexity of the interactions, study design and analysis issues apply, making it difficult to evaluate the impact of individual or combined measures [14]. One modelling study estimated that the combination of reduced class density, transmission mitigation measures (mask wearing, desk shields, frequent surface cleaning, outdoor teaching), and early identification of active infections would reduce SARS-CoV-2 prevalence [15]. Additionally, a study in Barcelona, Spain, found that compared to the general population, transmission rates of SARS-CoV-2 were lower among children attending summer schools that applied IPC measures such as contact bubbles, hand hygiene, mask wearing and outdoor activities [16]. Similar findings were observed in primary and secondary schools in Switzerland [17]. Given the limited evidence of the implemented of IPC measures at schools on SARS-CoV-2 transmission among school-age children and the potential impact of these measures on pupil’s wellbeing, a thorough evaluation of their impact on SARS-CoV-2 transmission in school environments is warranted.

To our knowledge, no information on the implementation of IPC measures against SARS-CoV-2 transmission in Belgian schools has been published thus far. Even though this manuscript reports on data from school year 2020–2021, the findings reported are still of interest. Beginning June 2022, of the 270,000 PubMed hits for COVID-19, less than 150 hits were found adding search terms such as IPC and schools. The objective of this paper is to document the implementation of IPC measures in Belgian primary and secondary schools and to assess how the implementation of such measures relates to the prevalence of anti-SARS-CoV-2 antibodies among pupils and staff.

## Methods

This analysis is based on data collected through a country-wide representative longitudinal prevalence study on SARS-CoV-2 antibodies among Belgian pupils and school staff during the school year 2020 – 2021 [18].

### Study design

Data on the implementation of IPC measures were collected from schools at the start of the study in December 2020/January 2021, and again in March and May/June 2021. At these three data collection periods, saliva samples were taken from (the same) pupils and staff to determine the prevalence of anti-SARS-CoV-2 antibodies. A descriptive analysis of the implementation of IPC measures in Belgian schools is presented for each of the three data collection periods, but the association between the strength of implementation of IPC measures and the SARS-CoV-2 seroprevalence among pupils and staff, was only assessed in the data collected in December 2020/January 2021.

### Study population and recruitment 

Schools, pupils and staff were recruited using a two-stage randomized cluster design with proportional allocation by province and sociodemographic background. In the first stage, 41 clusters were identified in which one primary school and one secondary school were selected at random from a list of all Belgian schools providing general education. Clusters were allocated per province proportional to the child population on January 1st 2020 [19]. In the second stage, among eligible participants who agreed to participate in each school, a random sample of 20 pupils and 10 staff was drawn.. Inclusion criteria were being a pupil attending the 2^nd^ or 3^rd^ grade (ages 7 to 9 years) of primary school, or a pupil attending the 2^nd^ grade (ages 13 to 14 years) of secondary school, or a staff member in contact with eligible pupils.

### Sample size

A sample size of 800 pupils and 400 staff at each school level (primary and secondary schools) was calculated to estimate the seroprevalence with a margin of error of 2.3% for pupils and 3.0% for staff, assuming a seroprevalence of 6% among pupils and 10% among staff and a cluster design effect of two. All participants were recruited during the first data collection period (December 2020/January 2021).

### Data collection 

The local study coordinator – a staff member of the participating school – collected the data on the implementation of IPC measures via a secured online questionnaire using the ‘LimeSurvey’ platform (LimeSurvey version 3.22.24 + 2,000,630; see Supplement I). The questionnaire was available in Dutch and French. The list of IPC measures assessed in the questionnaire was based on the recommendations announced in November 2020 by the Belgian education authorities [20, 21]. These recommendations included guidelines on school closures, school and classroom ventilation, personal and environmental hygiene and physical distancing. The baseline questionnaire provided information regarding the period between the reopening of schools (May 2020) and the first data collection period (December 2020/January 2021). The follow-up questionnaires provided information regarding the period between the previous and present data collection period.

During each data collection period, the prevalence of anti-SARS-CoV-2 antibodies among the same pupils and staff of the participating schools was assessed using saliva samples. This technique was validated in a pilot study in the province of Limburg [22]. These samples were self-collected via an Oracol device (Oracol, Malvern Medical Developments, UK) under the supervision of a trained nurse. Semi-quantitative levels of anti-RBD (Receptor Binding Domain) IgG were determined at the Immunology Laboratory of Sciensano (Public Health Belgium) using the WANTAI SARS-CoV-2 IgG ELISA (Quantitative) (Beijing, Wantai Bio-Pharm, China, cat n° WS-1396) customized for saliva samples using an in-house protocol. Assay performance and a specificity-optimized cut-off of > 1.5 signal-to-noise ratio for anti-RBD IgG positivity in saliva was evaluated using receiver operating characteristic analyses. Using this cut-off, the specificity of the test was 96.7% and 96.5% and the sensitivity was 95.1% and 80.0% for adults and children, respectively.

### Implementation of IPC measures 

The implementation of IPC measures in Belgian schools is presented using data from three data collection periods (December 2020/January 2021, March 2021 and May/June 2021).

IPC measures were grouped according to the target age as: [1] IPC measures applied in both, primary and secondary schools, [2] IPC measures applied in primary schools only, and [3] IPC measures applied in secondary schools only. Group 1 was further divided in to four subcategories: [1] school/class closures, [2] ventilation measures, [3] hygiene measures (environmental & personal), and [4] physical distancing measures (Table 1). We based this classification on the first ECDC technical report on schools [13], with an additional subcategory for ventilation measures.

For each data collection period, we reported the number and proportion of schools that implemented each IPC measure. For school and class closures, we reported the number and proportion of schools with at least one closure during the assessed period. The other IPC measures were assessed using a 5-point Likert scale ranging from 1 (the IPC measure was ‘not applied at all’) to 5 (the IPC measure was ‘fully applied‘). For the analysis, we considered measures with a score of 4 or 5 as ‘implemented’ and with a score from 1 to 3 as ‘not implemented’. When a school did not respond for a specific measure (Table S2, S3, Supplement II), we considered it as ‘not implemented’. The implementation of IPC measures was analysed at school level (primary versus secondary schools), data collection period (December 2020/January 2021, March 2021, May/June 2021) and language network level (French versus Dutch).

### Relation between the implementation of IPC measures and prevalence of anti-SARS-CoV-2 antibodies

Based on the data collected during the first data collection period (December 2020/January 2021), we assessed whether the implementation of IPC measures was associated with the prevalence of anti-SARS-CoV-2 antibodies among pupils and staff in primary and secondary schools. Only schools that provided an answer for at least 10 out of 14 measures were included in this analysis. Furthermore, in order to have a scale based on uniformly registered and comparable information, measures that were not included in both primary and secondary schools were not included. We also excluded measures related to school or class closures, as the reverse causality of school closures and cases cannot be assessed in this study.

Schools were classified according to their compliance with the implementation of IPC measure as ‘poor’, ‘moderate’ or ‘thorough’. This classification was based on the sum of Likert scale scores (each ranging from 1 to 5) for individual IPC measures. Schools in the upper quartile were designated as ‘thorough’, and schools in the lower quartile as ‘poor’ implementers. Schools with a sum of scores in between were considered ‘moderate’ implementers. If the Likert score was missing for a particular IPC measure in a school, we replaced the missing score by the mean score for that particular IPC measure from the other schools. If more than four measures were missing from the same school, it was not included in the analysis. For each of these three IPC measure compliance groups, the prevalence and 95% confidence interval (95% CI) of anti-SARS-CoV-2 antibodies among pupils and staff was calculated using generalized estimation equations to account for possible clustering of cases in schools. Generalized estimation equations for binomial outcomes with a log link function were used to assess if the implementation of IPC measures (poor, moderate, thorough) was associated with the prevalence of anti-SARS-CoV-2 antibodies, taking into account the community exposure (total reported cases in the school district 14 days before testing), socioeconomic tertile and language network (Dutch, French) of the school, type of school (primary, secondary), subject category (staff, pupil; when applicable), and school identification as the clustering variable with an exchangeable correlation structure. Results are expressed as an adjusted Relative Risk (aRR) with 95% CI. This analysis was done for all IPC measures together and for the IPC subcategories (ventilation, hygiene and physical distancing). We used data from the first collection period for this assessment, as this provided the most complete data (for example, during the second period less ventilation measures were assessed and for the third data collection period seroprevalence data were impacted by vaccination among staff).

Data were analysed using SAS Enterprise Guide version 7.1 (SAS Institute Inc., Cary, NC, USA) and R version 4.0 (2021, R. Foundation for statistical computing, Vienna, Austria).

### Ethics approval

The study was approved by the Medical Ethics Committee of the University Hospital Ghent (reference: B6702020000744—BC-08564). Before enrolment, written informed consent was obtained from staff and parents of participating pupils, and informed assent was obtained from pupils.

## Results

### Participating schools

We contacted 98 primary and 108 secondary schools of which 44 primary and 40 secondary schools agreed to participate. Of these 84 schools, 45 belonged to the Dutch and 39 to the French language network. All but one school (*n* = 83) completed the online questionnaire in December 2020/January 2021 (first data collection period) and 82 schools completed it in March and May/June 2021.

Eighty-one schools (43 primary and 38 secondary) fulfilled the requisite 10 (out of a total of 14) assessments of IPC measure implementation and were included in the analysis. Among these, 21 (26%) schools were classified as implementing the measures thoroughly, 37 (46%) as moderately and 23 (28%) as poorly. The mean prevalence of anti-SARS-CoV-2 antibodies for each group was calculated based on a total of 1,285 pupils (710 primary, 575 secondary school pupils) and 818 staff (432 primary, 386 secondary school staff). More details on sample recruitment and participation can be found in Figure S4 (Supplement III).

### Implementation of IPC measures

Table 1 shows the number and proportion of schools that implemented each of the individual IPC measures during the three data collection periods. For most IPC measures, similar implementation in primary and secondary schools was observed. Between the reopening of schools in May 2020 and the last data collection period in May/June 2021, 13 (16%) schools closed temporarily due to COVID-19. Hygiene measures were implemented most frequently, followed by physical distancing and ventilation measures. Overall, the implementation of individual IPC measures did not change substantially over the three data collection periods. Between March and May/June 2021, school closures were more frequent in primary than in secondary schools (Table 1).

**Table 1:** Number and percentage of Belgian primary and secondary schools that implemented IPC measures^a^ during December 2020/January 2021, March 2021 and May/June 2021^b^

Specific IPC measure	Primary schools			Secondary schools		
	M0 *N* = 43 *n* (%)	M3 *N* = 44 *n* (%)	M6 *N* = 43 *n* (%)	M0 *N* = 40 *n* (%)	M3 *N* = 38 *n* (%)	M6 *N* = 39 *n* (%)
**IPC measures applied in both primary and secondary schools (16 measures)**						
**Closures (2 measures)**						
School closure outside holiday breaks	1 (2)	3 (7)	7 (16)	0 (0)	3 (8)	1 (3)
Classes suspended	23 (53)	19 (43)	18 (42)	22 (55)	13 (34)	10 (26)
Ventilation measures (4 measures)						
Classrooms have a CO_2_ detector	9 (21)	/	11 (26)	3 (8)	/	4 (10)
School has and uses a ventilation system	9 (21)	/	4 (9)	5 (13)	/	5 (13)
Teachers are encouraged to ventilate class rooms regularly	41 (95)	42 (95)	40 (93)	39 (98)	38 (100)	35 (90)
Classes take place outside as much as possible	3 (7)	/	5 (12)	2 (5)	/	6 (16)
**Hygiene measures (environmental & personal) (5 measures)**						
Classrooms are cleaned regularly and more frequently than previous school years	26 (60)	31 (70)	31 (72)	27 (68)	32 (84)	29 (74)
Staff rooms are cleaned regularly and more frequently than previous school years	28 (65)	33 (75)	32 (74)	25 (63)	33 (87)	29 (74)
Toilets are cleaned regularly and more frequently than previous school years	31 (72)	40 (91)	37 (86)	31 (78)	38 (100)	31 (79)
Surfaces that are touched regularly are disinfected daily	33 (77)	33 (75)	33 (77)	33 (83)	30 (79)	26 (67)
Alcohol gel (or additional possibilities to clean hands) is made available for pupils and staff	39 (88)	42 (95)	40 (93)	38 (95)	37 (97)	37 (95)
**Physical distancing measures (5 measures)**						
Breaks are spread to decrease contact between different age groups	12 (28)	16 (36)	13 (30)	18 (45)	17 (45)	9 (23)
Number of staff is limited per room	32 (74)	35 (80)	35 (81)	33 (83)	30 (79)	29 (74)
Pupils have one fixed place in a fixed classroom	25 (58)	32 (73)	31 (72)	28 (70)	30 (79)	31 (79)
Teachers change between classrooms, not the pupils	22 (51)	31 (70)	27 (63)	28 (70)	28 (74)	28 (72)
Lunches are taken in the classroom. If this is not possible pupils have a fixed place in the dining area	36 (84)	42 (95)	38 (88)	34 (85)	35 (92)	30 (77)
**IPC measures applied in primary schools only (3 measures)**						
Staff wear a mask if sufficient distance cannot be maintained	34 (79)	43 (98)	39 (91)			
Distance is kept during contacts between adults	38 (88)	39 (89)	34 (79)			
Distance is kept during contacts between staff and pupils	24 (56)	18 (41)	24 (56)			
**IPC measures applied in secondary schools only (2 measures)**						
Secondary schools only: Staff and pupils always wear a mask inside				37 (93)	36 (95)	33 (85)
Secondary schools only: Staff and pupils wear a mask outside unless they can keep sufficient distance				36 (90)	37 (97)	33 (85)

^a^All measures, except for closures, are scored on a scale ranging from 1 (not applied at all) to 5 (fully applied). Measures with a score of ‘4’ or ‘5’ are considered ‘implemented’ by the school

^b^Schools that did not answer to certain measures were considered to not apply these measures. The number of missing school answers are given in Table S2, Supplement II

*IPC* Infection prevention and control, *M0* Data collection period at study month 0, 3 December 2020—28 January 2021, *M3* data collection period at study month 3, 1—26 March 2021, *M6* data collection period at study month 6, 17 May – 11 June 2021, *n* (%): absolute number of schools (percentage of schools) that implemented the measure, *N* number of schools that completed the questionnaire

Teacher﻿s﻿ were systematically encouraged to ventilate classrooms (93% of primary and 90% of secondary schools by May/June 2021), but notably fewer schools invested in the use of CO_2_ detectors (26% of primary and 10% of secondary schools by May/June 2021) or active ventilation systems (9% of primary and 13% of secondary schools by May/June 2021). Classes were rarely organized outdoors. Physical distancing measures were implemented in the majority of schools, except for the separation of age groups during breaks (30% of primary and 23% of secondary schools by May/June 2021). Findings from May/June 2021 indicated that pupils from 72% of secondary schools stayed in the same classroom instead of changing classrooms as usual. Most hygiene measures were implemented by more than 75% of schools during the three data collection periods. Mask wearing among staff of primary and secondary schools and pupils of secondary schools was reported to be well implemented (more than 80% compliance) (Table 1).

Implementation of IPC measures was overall similar in both language networks (Table S1, Supplement II). An exception is the use of fixed classrooms for pupils, which was more common in the Dutch language network (89% of schools) compared to the French language network (59% of schools) in May/June 2021. The opposite was noticed for fixed classrooms for teachers (49% Dutch and 82% French language network).

### Relation between IPC measures implementation and prevalence of anti-SARS-CoV-2 antibodies among pupils and staff 

**Fig. 1:**
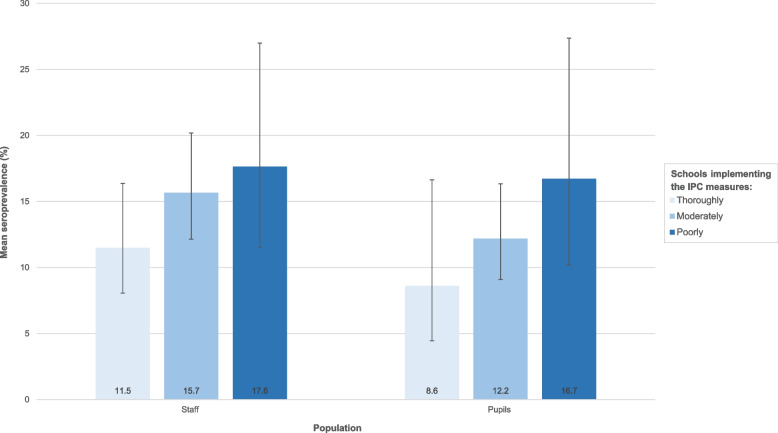
Prevalence of anti-SARS-CoV-2 antibodies according to the degree of IPC measure implementation Data collected at December 2020/January 2021 in Belgian schools among pupils and staff. The black lines indicate the upper and lower 95% confidence intervals. *IPC* Infection prevention and control

Figure 1 shows that, taking all IPC measures together, poorer implementation of these measures was associated with increased prevalence of anti-SARS-CoV-2 antibodies; observed as an increase from 8.6% (95% CI: 4.5 - 16.6) to 16.7% (95% CI: 10.2 - 27.4) among pupils and from 11.5% (95% CI: 8.1 - 16.4) to 17.6% (95% CI: 11.5 - 27.0) among staff. This association was statistically significant when considering pupils and staff together (aRR: 0.79, 95% CI: 0.64 - 0.98, *p* = 0.03), indicating a 21% decrease in seropositivity with thorough implementation of IPC measures. However, the association was not statistically significant for pupils (aRR: 0.77, 95% CI: 0.53 - 1.10, *p* = 0.15) or staff (aRR: 0.81, 95% CI: 0.62 - 1.06, *p* = 0.12) separately.

**Fig. 2:**
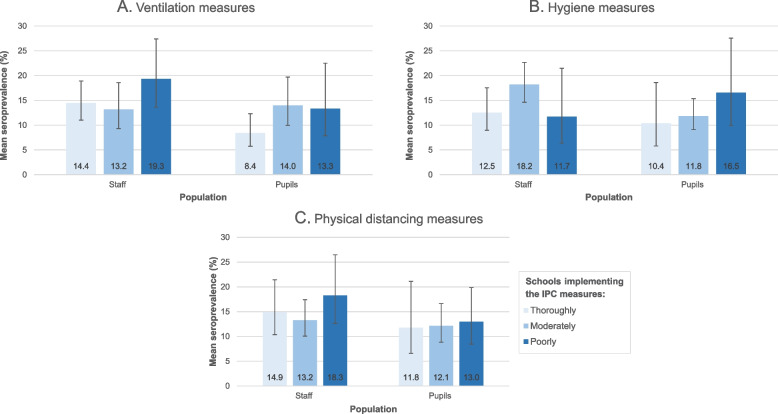
Prevalence of anti-SARS-CoV-2 antibodies according to the degree of subcategory IPC measures implementation. **A** Ventilation IPC measures, **B** Hygiene IPC measures, **C** Physical distancing measures Data collected at December 2020/January 2021 in Belgian schools among pupils and staff. The black lines indicate the upper and lower 95% confidence intervals. *IPC* Infection prevention and control

When this analysis was repeated for each subcategory of measures (Fig. 2), similar trends were observed in schools that implemented the measures poorly and thoroughly (except for the hygiene measures among pupils). However, none of these findings were found to be statistically significant (ventilation aRR: 0.96, 95% CI: 0.76 - 1.22, *p* = 0.76; hygiene aRR: 0.86, 95% CI: 0.69 - 1.07, *p* = 0.18; and physical distancing aRR: 0.90, 95% CI: 0.73 - 1.12, *p* = 0.35).

## Discussion

Apart from some ventilation and physical distancing measures, most IPC measures were implemented by more than 60% of primary and secondary schools. Overall, the implementation of IPC measures was similar in primary and secondary schools. However, since reopening in May 2020, more primary than secondary schools reported temporary closures due to the detection of COVID-19. Schools with poorer IPC measure implementation usually observed a higher prevalence of SARS-CoV-2 antibodies among pupils and staff. However, this association was only statistically significant for the assessment of all IPC measures together in the population compromised of pupils and staff. Despite observing comparable effect sizes in pupils and staff separately, statistically significant observations were not observed in analyses of neither these subpopulations nor the trends for the three different IPC measure subcategories.

The higher number of school closures observed in primary, compared to secondary schools, might be due to the general recommendation and infrastructure available for organizing distance learning in secondary, but not in primary schools. Secondary schools could more easily adjust to providing distance learning, while this option was more difficult to implement at primary schools. The general recommendation for secondary schools in Belgium was to organize distance learning for half of the school population for grades 3 and higher [12]. Although this recommendation did not apply to the secondary school pupils targeted in our study (2^nd^ grade), they limited the overall physical presence of pupils and staff in schools. Yet, the effect of school closures on the seroprevalence is less clear, largely because of the overlap with other IPC measures. A study from Norway found that compared with the implementation of targeted IPC measures in schools, school closures did not have an impact on the number of SARS-CoV-2 infections among pupils [23]. However, a study from Sweden found that in the beginning of the COVID-19 epidemic, exposure to open schools (implementing mild IPC measures) resulted in doubling of the infection rate among teachers and a small increase in infections among pupils’ parents, when compared with closed schools [24].

Most schools reported thorough application of hygiene measures, possibly as a result of the widespread attention for hand hygiene, disinfection and cleaning from the onset of the pandemic [25]; and because these measures are usually easy to implement. Ventilation measures were less frequently applied, perhaps because some, such as the installation of CO_2_ detectors or ventilation systems, require a higher financial investment, while measures like passive ventilation may be challenging during winter. In the category of physical distancing measures, staggering break times by age group was less often implemented, potentially due to logistic and infrastructural challenges. The decision to adjust our analysis for language network is related to differences observed in the two main Belgian regions; Flanders (Dutch speaking) and Wallonia (French speaking). For example, differences in regional authority policies, behaviour, culture and population density. Between the language networks, we did not observe a big difference in IPC measure implementation. However, in general, physical distancing measures were more frequently applied in schools from the Dutch language network which reflects differences in regional policies, school organization and regulations [12].

Mask wearing was reported to be almost fully implemented by primary and secondary school staff and by secondary school pupils. Although we could not assess the effect of mask wearing on SARS-CoV-2 prevalence in our study, findings in kindergarten and primary schools in Georgia (USA) suggest that this influences the transmission of SARS-CoV-2 in school settings [26]. Also, a report on a SARS-CoV-2 outbreak in a primary school in California (USA) revealed higher SARS-CoV-2 transmission among pupils sitting closer to the teacher who did not wear a mask and was identified as the index case [27].

Our study identified a statistically significant association between the prevalence of anti-SARS-CoV-2 antibodies and the implementation of IPC measures for the population compromised of pupils and staff together but not for pupils and staff separately. Since effect sizes, expressed as aRR, are of the same magnitude in both groups, this is probably the result of a loss of statistical power. Compliance with recommendations regarding IPC measures might thus be beneficial for the total school population. Results for the different subcategories of IPC measures point in the same direction, but effect sizes are notably smaller and not statistically significant. Studies do suggest that ventilation measures might be more effective [26], but more likely a combination of measures is needed to reduce the transmission of SARS-CoV-2 as illustrated by the ‘Swiss-Cheese Model’ [28]. Similarly, a study in the USA indicated that the risk associated with in-person teaching decreases when more IPC measures were implemented [29]. Although a combination of measures subcategories would work best to prevent the spread of SARS-CoV-2, it is important to apply only those that would be most effective while taking into account the children’s educational needs and the staff’s general wellbeing.

A strength of our study is the random selection of a geographically and socially proportional sample of pupils and staff from Belgian schools. Also, this study is the first to assess the implementation of IPC measures in schools in relation to the prevalence of anti-SARS-CoV-2 antibodies among pupils and staff in Belgium. There are also limitations. The data on the implementation of IPC measures were self-reported which is prone to information bias and misinterpretation. Additionally, data collection started in December 2020 and covered the preceding months which could result in recall bias. Moreover, the first testing period covered a rather heterogeneous phase of the pandemic including periods of in-person teaching and the summer holiday in July and August 2020. Another limitation is that the individual characteristics of the participants were not included in our analyses, and that the reverse causality of outbreaks and implementation of IPC could not be addressed. Furthermore, we adjusted for community exposure in our analysis of the impact of IPC measures on the prevalence of anti-SARS-CoV-2 antibodies in schools. This might have impacted the estimated effect of the IPC measures if the community prevalence was affected by the IPC measures.. In addition, we did not adjust the seroprevalence for test specification (sensitivity and specificity). Finally, while representative for Belgian schools, the sample sizes at the school level are small and do not allow to quantify the attributable impact of individual IPC measures.

## Conclusion

Most Belgian primary and secondary schools of both language networks complied relatively well with recommended IPC measures. Poor implementation of all such IPC measures together and separately, by ventilation, hygiene and physical distancing subcategory, showed an increase in the prevalence of anti-SARS-CoV-2 antibodies among pupils and staff in Belgian schools. This association was statistically significant for all IPC measures together in the population compromised of pupils and staff, with thorough IPC measures implementation associated with a 21% decrease in seropositivity. Despite observing comparable effect sizes in the assessment of pupils and staff separately, these did not reach statistical significance, and neither did the trends for the three different IPC measures subcategories. However, this study underlines the potential beneficial effect of IPC measures in reducing the spread of SARS-CoV-2 in a school setting.

## Supplementary Information

**Additional file 1.**

## Data Availability

The data used in the current study are not publicly available due to privacy concerns, but data that support the current findings are available from the corresponding author on reasonable request.

## Abbreviations

SARS-CoV-2: Severe acute respiratory syndrome coronavirus 2
WHO: World Health Organization
COVID-19: Coronavirus disease 2019
IPC: Infection prevention and control
UNESCO: United Nations Educational, Scientific and Cultural Organization
UNICEF: United Nations Children's Fund
IFRC: International Federation of Red Cross and Red Crescent Societies
RBD: Receptor Binding Protein
ELISA: Enzyme-linked immunosorbent assay
aRR: Adjusted Relative Risk
CI: Confidence Interval
